# Plasma p-tau231 and NfL differently associate with functional connectivity patterns in cognitively unimpaired individuals

**DOI:** 10.1007/s11357-025-01743-1

**Published:** 2025-06-19

**Authors:** Alejandra García-Colomo, David López-Sanz, Martín Carrasco-Gómez, Federico Ramirez‑Toraño, Soraya Alfonsín, Carlos Spuch, María Comis-Tuche, Fernando Maestú

**Affiliations:** 1 Center for Cognitive and Computational Neuroscience, Complutense University of Madrid, 28223 Pozuelo de Alarcón, Spain; 2 Department of Experimental Psychology, Cognitive Psychology and Speech & Language Therapy, Complutense University of Madrid, 28223 Pozuelo de Alarcón, Spain; 3 Department of Electronic Engineering, Universidad Politécnica de Madrid, 28040 Madrid, Spain; 4 Translational Neuroscience Research Group, Galicia Sur Health Research Institute (IIS-Galicia Sur), SERGAS-UVIGO, CIBERSAM, 36312 Vigo, Spain; 5 Health Research Institute of the Hospital Clínico San Carlos (IdISSC), 28240 Madrid, Spain

**Keywords:** Magnetoencephalograpy (MEG), Plasma biomarkers, P-tau231, NfL, Early biomarkers, Cognitively unimpaired, Alpha hyperconnectivity, Theta hypoconnectivity, Default mode network, Alzheimer’s disease continuum, Neurodegeneration

## Abstract

Amid the rising relevance of early and non-invasive markers for neurodegenerative diseases, such as Alzheimer’s disease, this study addresses the relationship between two promising candidates: electrophysiology and plasma markers. Specifically, functional connectivity, which underlies cognitive function, with p-tau231 (i.e., a marker of incipient Aβ pathology) and neurofilament light chain (NfL, i.e., a neurodegeneration marker) were considered. Seventy-five cognitively unimpaired individuals underwent a blood extraction and two magnetoencephalography recordings, approximately 3 years apart. First, correlation analyses were conducted to examine the association between the pathology markers’ concentration and FC. Subsequently, longitudinal FC changes were assessed, and their relationship with the markers’ concentration was evaluated. Default mode network regions, including the middle temporal gyrus, precuneus, hippocampus, and inferior parietal lobe, presented a positive association between their alpha FC and p-tau231 positively. In contrast, NfL levels were negatively associated with theta FC in frontotemporal regions. Moreover, these areas exhibit a longitudinal theta FC decrease. Importantly, the theta FC reduction was more pronounced as NfL concentrations increased. The described alpha FC alterations do not follow a maturation trajectory and are age-independent. Thus, the alpha FC increase associated with p-tau231 levels could constitute an early electrophysiological biomarker of Aβ pathology. In contrast, the longitudinal theta FC decrease, enhanced by NfL, could constitute an early sign of neurodegeneration.

## Background

Alzheimer’s disease (AD) biological definition prompted the conception of the disease as a continuum, marked by an initial asymptomatic stage with emerging neuropathology [1]. Hence, identifying reliable markers that detect incipient pathological hallmarks has become a paramount research goal as it could provide a critical opportunity for disease-modifying interventions.

Positron-emission tomography (PET) and cerebrospinal fluid (CSF) have become key, as they allow for the detection of AD hallmarks at the different stages of the disease. PET tracers that detect Aβ and tau allow the visualization of the presence and location of these molecules, while CSF levels of different proteins are indicative of underlying pathology [2]. However, these techniques present important caveats, including limited accessibility, elevated costs, and invasiveness, which restrict their use in clinical practice as screening and disease-tracking tools [2, 3]. This has prompted a growing interest in blood-based biomarkers, which constitute a more accessible alternative to AD pathology detection. Phosphorylated tau at threonine 231 (p-tau231) is a recently discovered isoform of tau. Like other plasma species of phosphorylated tau, it does not seem to behave as a marker of tauopathy or strongly correlate with tau-PET, but rather shows a strong association with Aβ, especially in the early stages of the disease [1, 2]. This species shows alterations in plasma levels very early on in the continuum, parallel to those of soluble Aβ, even before Aβ-PET positivity, which tracks Aβ plaque deposition, has been achieved [4–8]. Likewise, this isoform shows a superior performance during incipient stages of the disease [9]. Therefore, it is considered a proxy of Aβ. On the other hand, neurofilament light chain (NfL) is a promising biomarker of neurodegeneration. Elevated levels of NfL are indicative of neurodegeneration-related axonal injury and have been reported elevated in the dementia, prodromal, and even preclinical stages of AD [3, 10]. Although it is not a specific marker of AD, elevated NfL levels are associated with a greater risk of AD dementia, brain atrophy, and AD progression and severity [2, 3, 11].

Likewise, electrophysiological techniques are becoming more relevant. These techniques directly measure neuronal activity, which shows incipient alterations as a result of the emergence and accumulation of AD pathology [12]. Moreover, electrophysiological techniques present additional advantages over other techniques, such as magnetic resonance or positron emission tomography, including minimal invasiveness and great temporal resolution [13].

Changes in functional connectivity (FC) have been reported throughout the continuum, associated with different hallmarks of the disease, stages, and regions. Regions that exhibit initial increases in Aβ also present increased connectivity [14–18]. The initial rise in FC associated with Aβ follows an inverted U-shaped pattern, giving way to a state of hypoconnectivity in the affected regions later on in the continuum, which is accompanied by a state of hyperconnectivity in previously intact regions [19–21]. It is thought that the Aβ-associated increase in connectivity is due to the positive feedback established between Aβ deposition and neuronal excitability, which underlies hyperconnectivity [15, 16, 22, 23]. In contrast, the decrease in connectivity observed throughout the continuum of AD, which has been classically considered a disconnection syndrome, has been associated with axonal pathology and neuronal death [15, 16].

Previous work from our group assessing cognitively unimpaired individuals reveal early electrophysiological alterations in association with p-tau231, which are consistent with an Aβ-induced excitation/inhibition imbalance. For instance, a significant association between the FC of the precuneus and plasma p-tau231 and centrality shifts congruent with incipient underlying Aβ pathology were found [24–26]. Building on this evidence, in the current paper we aimed to study, for the first time, the relationship between different markers of pathology (i.e., NfL and p-tau231) and whole-brain FC in cognitively unimpaired subjects. Thus, in this study, we follow a data-driven protocol, without regions or frequency bands of interest. As p-tau231 is considered a proxy of Aβ pathology due to their temporal and disease-specific association, we expect to find positive associations between p-tau231 levels and FC. Conversely, considering NfL’s nature as a neurodegeneration marker, we expect to find negative associations with FC. Moreover, we aim to study the longitudinal evolution of the connectivity measures and address the relationship between the FC change and the neuropathology markers.

## Methods

### Participants

This study is framed within a larger initiative, aimed at tracking the longitudinal evolution of a group of cognitively unimpaired participants over the years to detect the earliest electrophysiological signs of Alzheimer’s disease pathology. As a result, the data for the current study were obtained from two assessments (i.e., baseline and follow-up), approximately 3 years apart, both funded by the Spanish government. The present study used MEG recordings and structural MRI images from the baseline assessment. On the other hand, MEG recordings, plasma determinations, and a neuropsychological evaluation to corroborate their cognitive status, from the follow-up assessment, were used. This evaluation included the Montreal Cognitive Assessment [27], the Trail Making Test [28], the word list test, and digit backward span subscales from the Wechsler Adult Intelligence Scale III [29] and the Rey-Osterrieth complex figure [30]. MRI images were only available for the baseline assessment, whereas plasma determinations were only available for the follow-up.

The sample for this study consisted of 75 individuals, all cognitively unimpaired, who had available plasma p-tau231 and NfL determinations and a valid MEG recording at follow-up (Table 1), and an available and valid MRI scan from the baseline assessment. Seventy-one of these individuals had available and valid MEG recordings from the baseline assessment.

**Table 1:** Demographic information of the sample. The p-tau231 is presented in pg/ml, whereas the NfL is presented in ng/ml. MoCA, Montreal Cognitive Assessment; TMT, Trail Making Test, versions A and B; Digits-b, digits backward; WLT, word list test, (t) total immediate recall and (d) delayed recall; Rey, Rey-Osterrieth complex figure, (i) immediate and (d) delayed recall

	Mean (std)
M/W	23/52
Age	60.573 (5.973)
p-tau231	384.889 (151.245)
NfL	0.973 (1.253)
MoCA	28.213 (1.328)
Dig-f	6.456 (0.927)
Dig-b	4.772 (1.181)
TMT-A	31.421 (10.445)
TMT-B	68.736 (29.527)
WLT-t	35.246 (5.214)
WLT-d	8.982 (1.959)
Rey-i	20.430 (6.824)
Rey-d	19.903 (6.193)

The exclusion criteria for the present study were as follows: (1) history of psychiatric or neurological disorders; (2) family history of dementia other than Alzheimer’s disease; (3) signs of infection, infarction, or focal lesions in the T2-weighted MRI scan; (4) alcoholism or chronic use of anxiolytics, neuroleptics, narcotics, anticonvulsants, or sedative-hypnotics; (5) being under 50 or above 80 years of age; (6) cognitive impairment; (7) unusable follow-up MEG recording; (8) unavailable or unusable baseline T1-weighted image; and (9) unavailable plasma p-tau231 or NfL determination.

This study was approved by the “Hospital Clínico San Carlos” Ethics Committee and complied with internationally accepted guidelines and regulations.

### Plasma determination

Plasma concentrations of the proteins p-tau231 and NfL were quantified by competitive enzyme-linked immunosorbent assay, using commercial kits (MyBiosource, Inc., USA, MBS724296, and human NF-L Elabscience, respectively), following the manufacturers’ instructions. Duplicates were performed for all subjects. An automated microplate reader (Biochrom ASYS UVM 340, Cambridge, UK) measured the optical density at 450 nm with Mikrowin 2000 software (Berthold Technologies, Germany).

### MEG recordings and preprocessing

Four minutes of eyes-closed resting state brain activity was recorded from every participant using the 306-sensor Vectorview MEG system (Elekta AB, Stockholm, Sweden) located at the Center for Biomedical Technology (Madrid, Spain). Participants’ heads were digitized with a Fastrack Polhemus system (Polhemus, Colchester, VT, USA), which was also used to determine the position of four head-tracking coils, placed at the mastoids and the foreheads of participants. Additionally, electrooculographic and electrocardiographic activity was recorded using two sets of bipolar electrodes. Recordings were carried out inside a magnetically shielded room, where participants were instructed to remain still and relax. Data were filtered online (0.1 to 330 Hz) and acquired with a 1000 Hz sampling rate. Head movements and external noise components were removed with the temporal expansion of the signal space separation method [31], implemented by Neuromag Software (MaxFilter version 2.2, correlation limit 0.90, time window 10 s).

Next, MEG signal was preprocessed. An MEG expert, blind to participants’ information, removed physiological and jump artifacts with Fieldtrip [32]. Then, cardiac activity and blink components were removed using an ICA-based algorithm. Data were divided into segments of 4 consecutive seconds of artifact-free activity. Forty-five segments were randomly selected for each subject and kept for further analyses to ensure an equal number of trials for all the participants to estimate FC. Subsequent analyses only used data from magnetometers due to the redundant nature of the data after applying the signal space separation method [33].

### Source reconstruction

Source-space MEG signal was reconstructed using each individual’s T1 brain MRI. MRI scans were performed with a General Electric 1.5 T system equipped with a high-resolution antenna and a homogenization-phased array uniformity enhancement filter (fast spoiled gradient echo sequence, TR/TE/TI = 11.2/4.2/450 ms; flip angle 12°; slice thickness 1 mm, 256 × 256 matrix and FOV 25 cm).

The source model consisted of 2459 sources, arranged in the three-dimensional 1-cm grid defined by the Montreal Neurological Institute template. This model was linearly transformed into subject space using each participant’s individual T1-weighted image. Single shell models were generated using SPM12 brain segmentation [34]. The head model, the source model data, and a modified spherical solution were combined to calculate the lead field matrix.

MEG data were filtered between 2 and 45 Hz using a 450th-order finite input response filter designed using a Hann window. Data were filtered using a two-pass approach to avoid phase distortion. A total of 2000 samples of real data were placed at the beginning and end of the signal as padding to avoid edge effects. Linearly constrained minimum variance beamformer was employed to solve the inverse problem. Each source position was labeled using the automated anatomical labeling atlas [35].

### Functional connectivity analysis

Sources’ time series were band-pass filtered in the delta (2–4 Hz), theta (4–8 Hz), alpha (8–12 Hz), beta (12–30 Hz), and gamma (30–45 Hz) frequency ranges. FC between all pairs of sources was estimated using amplitude envelope correlation with leakage correction (AEC-c) for all 45 epochs of each subject. This procedure first performs a symmetric pairwise orthogonalisation of the signal in order to remove zero-lag correlations, which could result from spurious signal coupling due to volume conduction (i.e., signal leakage in source-space). Then, the AEC-c is calculated as the absolute value of the Pearson correlation between time-series’ envelopes. Multiple studies have shown the reliability of AEC-c as a metric of FC [36, 37]. This results in five AEC-c adjacency matrices that contain the FC values between all pairs of sources in each frequency band.

### Statistical analysis

All statistical analyses were carried out in Matlab R2019b. In our first analysis, we assessed the relationship between FC in each frequency band and p-tau231 and NfL concentrations, all obtained at the same timepoint (i.e., the follow-up assessment). To avoid the spatial constraints imposed by analyses based on atlases, as well as overcome the multiple comparisons problem, typical of neuroimaging studies [38], we performed cluster-based permutation tests (CBPT) based on Spearman correlation analysis. This non-parametric tool returns clusters composed of neighbouring sources whose individual strength (i.e., weighted global FC) in a given frequency band shows a significant association with the neuropathology marker under study without a priori assumptions about specific sources and regions grouping.

To do this, first, a rho value for each source is obtained. The source-level significance threshold was set at 0.05. Next, a clustering procedure was performed, which considered groups of significant and spatially adjacent nodes, as well as the mass of each cluster (i.e., the sum of the rho values from the significant sources included in the cluster). Importantly, in order to avoid spurious findings, we set the minimum size of candidate clusters to 1% of the total sources; clusters smaller than this are considered non-significant. Lastly, to account for multiple comparisons, we performed 10,000 permutations, shuffling the neuropathology marker’s value, randomly reassigning a different marker’s value to each subject in each permutation. At each repetition, the maximum cluster mass statistic of the surrogate clusters was stored. This results in a distribution of the maximum cluster mass statistic, which allows us to compute the *P*-value for each candidate cluster of the original data. Cluster-level significance was set at 0.05.

This procedure was applied independently for each frequency band with each neuropathology marker. Additionally, these analyses were hypothesis-driven; since we expected to find positive associations between p-tau231 and FC, these CBPTs were right-tailed, while those performed with NfL were left-tailed. Importantly, all correlation analyses were performed using age as a covariate.

Next, we aimed to evaluate whether a longitudinal change in the FC of the clusters could be observed between the baseline and the follow-up MEG recordings. Lastly, the association between neuropathological concentrations and the total longitudinal change was addressed. To do this, we calculated the average strength of FC only for those clusters and bands showing significant association between FC and neuropathological markers’ concentration, both for the baseline and follow-up data. This returned two strength values, one for each cluster, per subject, corresponding to the baseline and follow-up FC evaluations. These values were statistically compared by means of an independent samples Student’s *t*-test.

Next, we aimed to evaluate whether this longitudinal change in FC of the defined clusters was associated with the pathology load. To do this, we calculated the FC change as:

$$\Delta \mathrm{FC}=\mathrm{follow}-\text{up cluster strength}-\text{baseline cluster strength}$$

This returned a single value of change per subject and cluster, which was then used to calculate the Spearman correlation with the pathology marker used to identify the cluster under study in the previous step. As no strong a priori assumptions were held in this case, a two-tailed analysis was performed, maintaining age as a covariate.

## Results

**Fig. 1:**
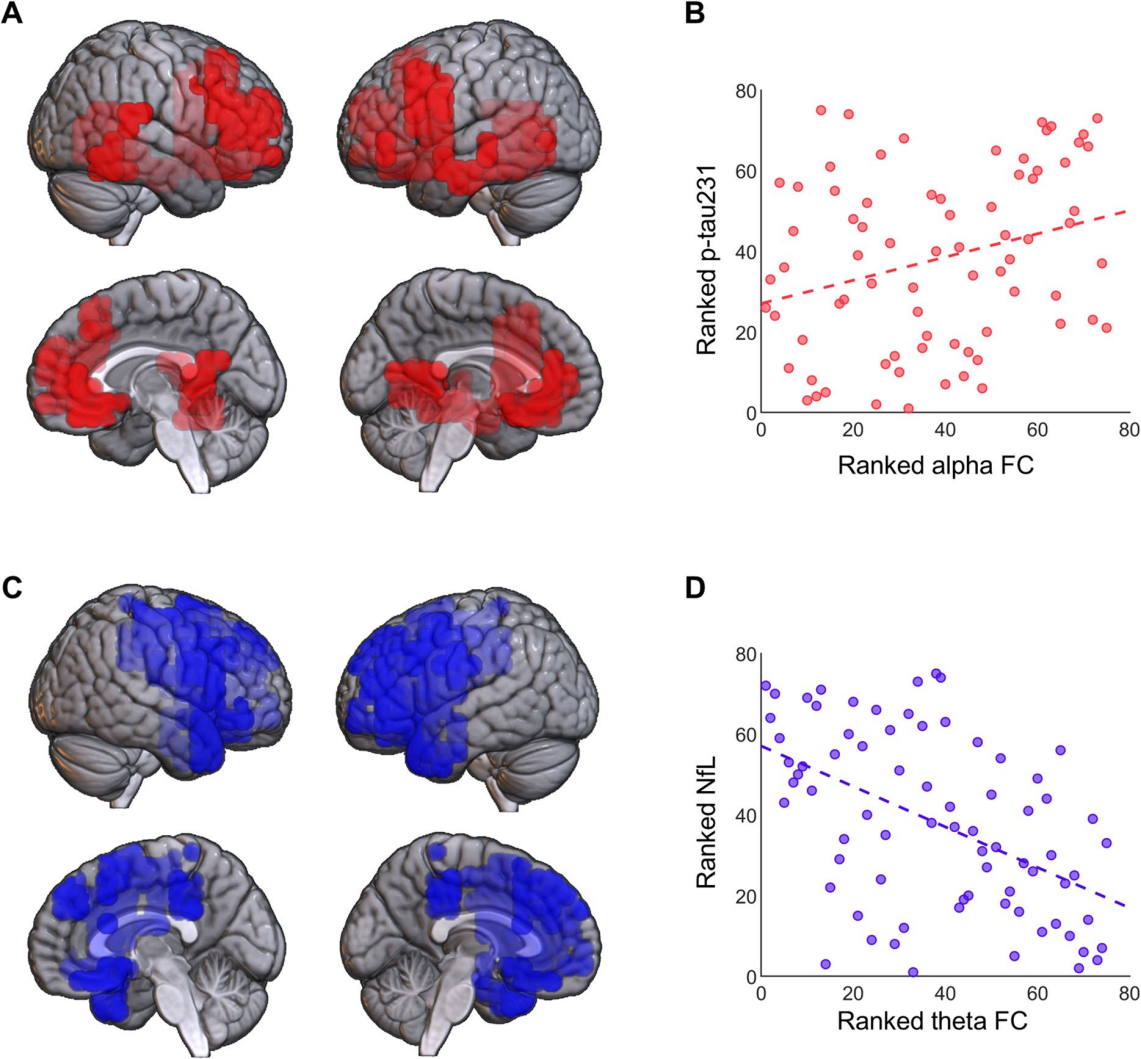
Significant clusters of FC association with plasma markers concentrations. **A** In red, a significant cluster comprising regions whose FC strength in alpha showed a positive correlation with plasma p-tau231 levels and **B** the scatter plot showing the association between the ranked alpha strength values and ranked p-tau231 values, obtained after regressing out the contribution of age on both variables. **C** In blue, a significant cluster encompassing regions whose FC strength in theta showed a negative correlation with plasma NfL concentrations and **D** the scatter plot showing the association between the ranked theta strength values and ranked NfL values, obtained after regressing out the contribution of age on both variables

The CBPT analyses performed to detect significant regional associations between FC and plasma concentrations of neuropathology markers returned two significant clusters, one for p-tau231 and another one for NfL (Fig. 1). The former was a positive cluster (rho_sum_ = 103.856, *p*_cluster_ = 0.048) composed by sources belonging to regions such as the precunei and posterior cingulate cortices, the hippocampal formation, the inferior parietal lobe, or the medial prefrontal cortex that showed a positive correlation (rho_average_ = 0.335, *p* = 0.004, *r*^2^ = 0.11) between their FC strength in alpha with p-tau231. On the other hand, a negative cluster was identified (rho_sum_ = −130.964, *p*_cluster_ = 0.005), encompassing mostly frontal regions and the temporal poles, bilaterally, where FC strength in theta negatively correlated with NfL plasma concentrations (rho_average_ = − 0.536, *p* = 8.610 × 10^−7^, *r*^2^ = 0.29). Regarding p-tau231, one more positive cluster was identified in the gamma band although it did not reach the significance threshold (rho_sum_ = 9.83, *p*_cluster_ = 0.24). In relation to NfL, five non-significant clusters emerged, one in the delta band (rho_sum_ = − 6.77, *p*_cluster_ = 0.32), one in the theta band (rho_sum_ = − 13.97; *p*_cluster_ = 0.167), and three in the gamma band (rho_sum_ = − 23.95, *p*_cluster_ = 0.11; rho_sum_ = − 23.11, *p*_cluster_ = 0.11; rho_sum_ = − 10.38, *p*_cluster_ = 0.23, respectively).

**Fig. 2:**
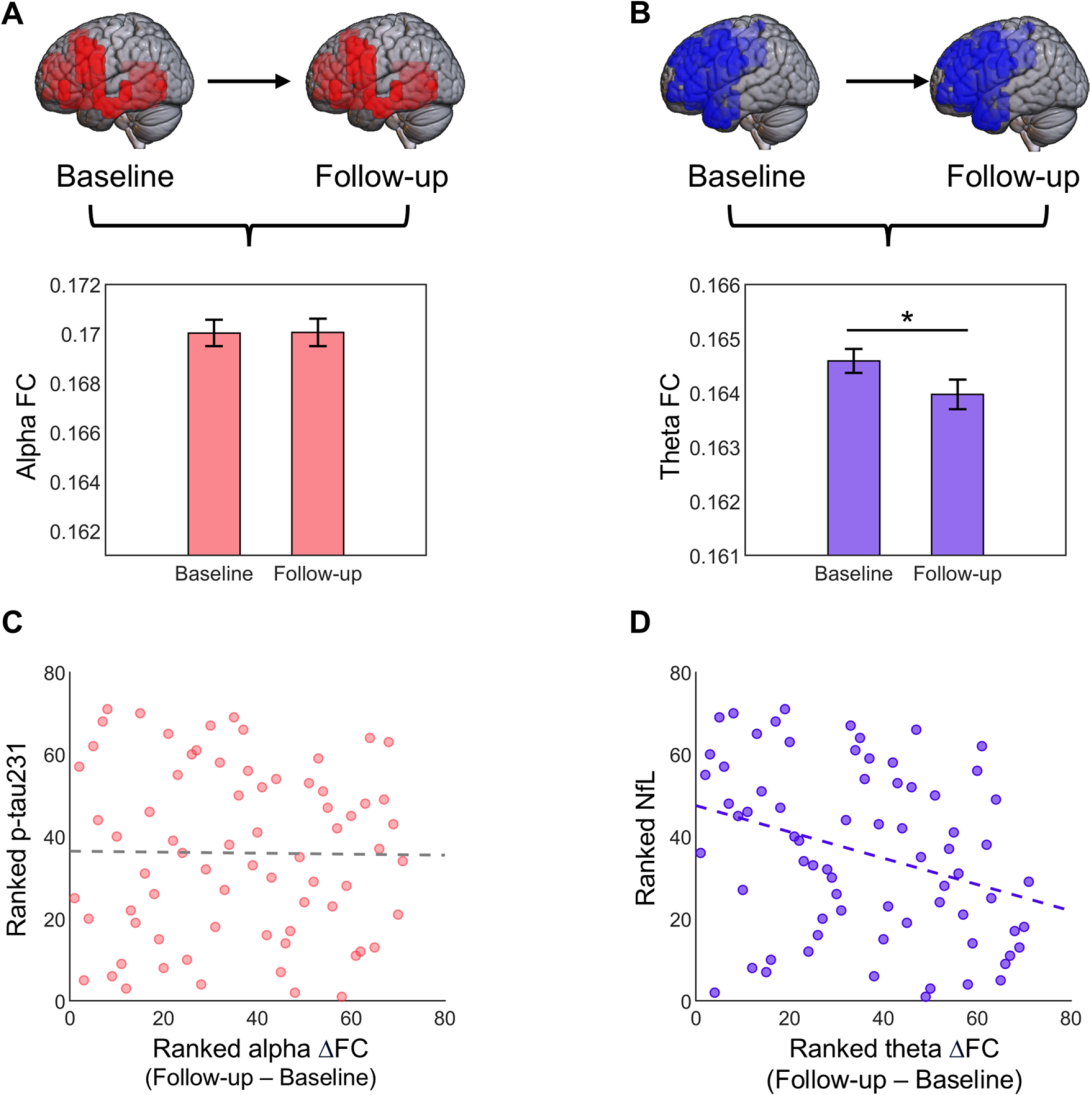
Longitudinal evolution of the clusters’ FC strength and its association with plasma pathology markers. **A** A bar plot showing no significant change in alpha FC strength over time in this cluster. **B** A bar plot where significant differences can be observed between the baseline and follow-up values of theta FC strength, which demonstrate a longitudinal FC reduction. **C** A scatterplot created with the ranked alpha FC strength values and ranked p-tau231 values, after eliminating the influence of age on either. No significant correlation can be observed. **D** A scatterplot showcasing the relationship between the change in FC strength and NfL levels. The scatterplot was created with the ranked values of FC and NfL, after controlling for the effect of age. Importantly, ΔFC values ranked lower reflect more negative values (i.e., larger FC longitudinal decrease)

In our following analysis we addressed the longitudinal evolution of the FC in the identified clusters. The average FC strength of the cluster identified in the alpha band in the previous analysis showed no significant differences between the baseline and follow-up evaluations (*t* = − 0.066; *p* = 0.947; Fig. 2A). On the other hand, the evolution of the cluster emerging in the theta band showed a significant difference between theta FC in the baseline and follow-up assessments (*t* = 2.058; *p* = 0.043; Fig. 2B), whereby a longitudinal reduction in theta FC strength was observed within the cluster.

Lastly, we evaluated whether the amount of longitudinal change in alpha and theta FC, within their respective clusters, was associated with the follow-up plasma concentrations of p-tau231 for the alpha cluster, and NfL for the theta cluster. Plasma p-tau231 levels did not show a significant association with the longitudinal FC change in the defined cluster (rho = 0.047; *p* = 0.702; Fig. 2C), as expected, since no significant longitudinal change was detected within this cluster’s FC in the first place. On the other hand, plasma NfL concentration showed a negative correlation with the change in theta FC within the defined cluster (rho =  − 0.358; *p* = 0.002; Fig. 2D). Since the longitudinal change was calculated as “follow-up FC − baseline FC” and a longitudinal FC decrease was detected, the values of FC change are mostly negative. Therefore, greater FC changes (i.e., more negative ΔFC values) are associated with higher NfL concentrations. Thus, larger longitudinal decreases in theta band FC within this cluster are associated with increased plasma NfL levels, regardless of age.

## Discussion

In this paper, we have found an association between two plasma pathology markers, one specific for AD pathology (p-tau231) and a non-specific one for neurodegeneration (NfL), and the connectivity patterns of cognitively unimpaired individuals measured with MEG. Our results suggest that both pathology markers are associated with early brain functional alterations, as manifested by the positive correlation observed between p-tau231 and alpha FC in regions such as the precunei and posterior cingulate cortices, the hippocampal formation or the medial prefrontal cortex, and the negative association observed between NfL and theta FC in frontal areas and temporal poles. Moreover, the influence of both time and NfL over decreasing theta FC suggests an exacerbation of the natural decrease in FC over time by pathological processes involving early neurodegeneration. On the other hand, the lack of longitudinal alpha FC change within the cluster and the lack of association between p-tau231 and alpha FC change are indicative of an incipient relationship between p-tau231 and alpha FC, which is not affected by age, thus making it a potential early functional biomarker providing spatial information.

Growing evidence from the last years has established plasma p-tau231 as an Aβ proxy, due to the close association between both markers, particularly in the early stages of the disease, even over other p-tau isoforms [1, 9]. Indeed, plasma p-tau231 presents abnormal levels even before Aβ-PET positivity is achieved and long before neurofibrillary tangles are detected with PET. Therefore, alterations in this marker can be considered a sign of incipient Aβ rather than of tau pathology and hyperphosphorylation [1]. Given the extensive literature that has linked incipient Aβ pathology to neuronal hyperexcitability and hyperconnectivity [15, 16, 39], we hypothesized a positive relationship between p-tau231 levels and FC. This was corroborated, as we found a positive correlation between p-tau231 and alpha FC, which was not explained by age, in a cluster that includes regions belonging to the default mode network, such as both precunei and posterior cingulate cortices, the hippocampi and parahippocampal cortex, the medial prefrontal cortex, and dorsolateral prefrontal cortex [40]. These results are consistent with previous research from our group, which revealed centrality shifts among unimpaired individuals in regions of early Aβ accumulation, associated with p-tau231 levels [25, 26]. Taken together, both sets of findings are congruent with an Aβ-dependent shift towards neuronal hyperexcitation, known to enhance FC and slow-frequencies’ centrality in the affected regions [41, 42]. Specifically, in the context of AD, these regions have been extensively studied due to the early histopathological, metabolic, and connectivity alterations they manifest. From a histopathological perspective, regions such as the precuneus and posterior cingulate cortex are among the first areas to exhibit enhanced Aβ secretion and deposition [16, 17]. From a network perspective, hubs belonging to the default mode network consistently exhibit enhanced vulnerability to AD pathology in empirical, computational, and theoretical models. As these regions show a higher metabolic consumption, which establishes a positive feedback loop with Aβ secretion, they are more vulnerable early on in the disease course, when they present enhanced connectivity [16, 42, 43].

The positive correlation between p-tau231 and greater alpha FC values is congruent with previous results that have demonstrated early alterations in this frequency band from the earliest stages of the disease. Most studies have focused on later stages where a peak slowing, a reduction in alpha power, and a decreased alpha FC have been described [19, 44–46]. Studies performed early on in the continuum, including among individuals at risk of developing the disease, show enhanced power, FC, and network reorganizations within this frequency band tied to the Aβ increases [19, 24, 26, 47–49]. Likewise, various studies have reported alpha connectivity increases among prodromal individuals prior to conversion to dementia [50, 51].

Alpha FC within this cluster remained stable over time, indicating no maturational changes within the studied age range and period. Moreover, the absence of correlation between FC change and p-tau231 concentrations, paired with the cross-sectional positive association between the follow-up alpha FC and p-tau231, could indicate that the functional alterations within this cluster in the alpha band are not attributable to age or normal longitudinal evolution. Instead, these alterations would be AD-specific and due to underlying pathology, which is emerging in some of the individuals of the sample. Therefore, the alpha FC within the defined cluster could constitute an early electrophysiological biomarker of AD pathology. In subsequent evaluations, we would expect a further increase in FC among individuals with already elevated p-tau231 concentrations that would, eventually, give way to a decrease in connectivity, thus mirroring the described trend of evolution in studies including prodromal converters to dementia [50, 51].

Multiple studies have associated neurodegeneration with states of hypoconnectivity. Through the AD continuum, after the initial rise in connectivity associated with Aβ pathology, a state of hypoconnectivity is described in regions that manifest neurodegeneration [19, 51]. On the other hand, tau pathology identified through tau-PET spatially correlates with decreased FC [16, 46, 52]. Importantly, tau-PET marks neurofibrillary tangles, which are tightly associated with neurodegenerative metrics and cognitive decline, and is known to appear later on than Aβ alterations [53, 54]. When both tau-PET and Aβ-PET positivity are present, even in unimpaired individuals, the effect of Aβ usually prevails and hyperconnectivity emerges [16]. In the context of AD, neurodegeneration and the reduction in connectivity are thought to be the result of chronic and extensive damage caused by the conjoined effect of prolonged excitotoxicity, Aβ toxicity, tau silencing, and tau hyperphosphorylation, which lead to axonal damage and, eventually, neuronal death [16, 53, 55]. Since NfL is a neurodegeneration marker, we hypothesized a negative association between FC and NfL in our sample. Our results support this hypothesis, as a negative correlation between NfL and theta FC was found in a cluster encompassing mostly the frontal region and temporal poles of the brain.

Previous studies performed with NfL have reported elevated concentrations throughout the continuum, but some discrepancies emerge in the earliest stages of the disease. Hu et al. [3] report elevated NfL levels among preclinical individuals, while Mattsson et al. [10] report no such alterations. These inconsistencies could stem from additional co-occurring pathological processes other than AD, driving the accelerated neurodegeneration not typically associated with emergent Aβ pathology [1, 54]. One of the factors more widely studied as responsible for this early neurodegenerative process is vascular pathology [56]. Indeed, neurovascular dysfunction is recognized as another hallmark of AD that manifests very early on and is influenced by multiple environmental, lifestyle, and genetic factors [57]. Notably, this vascular dysfunction can also occur in normally aging individuals and in individuals before they enter the AD continuum [58]. These individuals tend to present greater vascular damage in frontal regions, including greater reductions in blood flow or a greater proportion of white matter hyperintensities [59–61]. Additionally, some studies have reported alterations in theta activity in healthy aging and individuals with vascular problems. Vysata et al. [62] reported an age-related decrease in global theta in a sample over 17,000 individuals during resting state, while Xu et al. [63] reported a reduction in interhemispheric theta prior to stimulus presentation among individuals with vascular alterations. Babiloni et al. [64] and van Straaten et al. [65] reported a similar reduction in frontal FC for individuals with vascular decline during resting state.

Consequently, our cluster defined from the association between theta FC and NfL is congruent with vascular-mediated neurodegeneration, which, although not necessary for AD pathogenesis, is known to exacerbate it from the early stages. Moreover, the longitudinal reduction in theta FC within this cluster, appearing within the whole sample, suggests a normal age-dependent deterioration of these regions, consistent with the aforementioned age-related vascular worsening. Indeed, the negative association between greater theta FC reductions and NfL concentrations suggests that the aging-related FC decline is enhanced among individuals with elevated neurodegeneration. This is in agreement with the thesis posed by Jack et al. [1, 50], suggesting that neurodegeneration markers can be considered a sign of severity and predict further deterioration. Consequently, we would expect individuals with higher NfL levels to show greater FC alterations in future evaluations. It should be noted that, for the present study, no data to corroborate the vascular hypothesis was available. Future studies should include biomarkers destined to measure vascular function to address the relationship with NfL and brain function.

This paper unveils the early relationship between plasma pathology markers and brain function in cognitively unimpaired individuals, identifying FC patterns that could work as early biomarkers of AD pathology and neurodegeneration. However, some limitations should be considered. Future longitudinal assessments should incorporate established techniques alongside the plasma measures to strengthen the validity of these markers and the results obtained, as plasma biomarkers do not yet have diagnostic approval and the clinical implications from results should be considered with caution. Nevertheless, plasma biomarkers are emerging as a cost-effective, minimally invasive, and widely available alternative that is gaining support and validation. Additionally, baseline plasma biomarkers were unavailable, making the relationship between FC change and markers retrospective. This limited our ability to study the change in FC associated with the change in pathology markers and predict the FC evolution based on the baseline plasma markers concentrations. To overcome this, a new longitudinal evaluation has been approved and funded.

This study underscores the relevance of non-invasive techniques, such as MEG and plasma sampling, which complement each other, offering insight into the spatial distribution of early functional changes and information on the underlying pathological process. This complementary approach enables the prompt identification of individuals at risk of further neuropathology, such as the one caused by AD.

## Data Availability

The data that support the findings of this study are available from the corresponding author, upon reasonable request.
